# Comparative effectiveness of therapeutic ultrasound and pharmacotherapy in the management of muscular temporomandibular disorders: a randomized clinical trial

**DOI:** 10.3389/froh.2026.1923134

**Published:** 2026-08-27

**Authors:** M. Subhikshaa, Prathima Shetty, K. M. Veena, Prashanth Shenoy, Rachana Prabhu, Laxmikanth Chatra

**Affiliations:** Department of Oral Medicine and Radiology, Yenepoya Dental College, Yenepoya (Deemed to be University), Mangalore, Karnataka, India

**Keywords:** orofacial pain, pharmacotherapy, randomized clinical trial (RCT), temporomandibular disorders (TMDs), therapeutic ultrasound

## Abstract

**Introduction:**

Temporomandibular disorders (TMDs) of muscular origin are a common cause of chronic orofacial pain and functional limitation. Conservative management includes pharmacotherapy and physiotherapeutic modalities such as therapeutic ultrasound. However, evidence directly comparing their effectiveness remains limited. This randomized clinical trial compared therapeutic ultrasound and pharmacotherapy in reducing pain intensity and improving mandibular function in patients with muscular TMDs.

**Materials and methods:**

A prospective, parallel-group randomized clinical trial was conducted among 36 patients diagnosed with muscular TMDs according to Group I of the Research Diagnostic Criteria for Temporomandibular Disorders (RDC/TMD). Following ethical approval (YEC-1/2024/038) and CTRI registration (CTRI/2024/12/077788), participants were randomly allocated into two groups (*n* = 18). Group A received ibuprofen (400 mg), paracetamol (325 mg), and chlorzoxazone (250 mg) twice daily for seven days. Group B received therapeutic ultrasound (1 MHz, 1.0 W/cm², continuous mode) for 10 min daily for seven days. Pain intensity was assessed using the Numerical Rating Scale (NRS), and maximum mouth opening was measured using a Vernier calliper. Data were analysed using paired and independent Student’s t-tests (*p* < 0.05).

**Results:**

All participants completed the study. Both groups showed significant reductions in pain intensity and improvements in maximum mouth opening (*p* < 0.05). Mean NRS scores decreased from 6.31 ± 1.35 to 2.94 ± 1.06 in the pharmacotherapy group and from 5.56 ± 1.97 to 1.69 ± 1.08 in the therapeutic ultrasound group. Mean mouth opening increased from 40.31 ± 3.63 mm to 41.44 ± 2.94 mm and from 40.75 ± 3.55 mm to 43.63 ± 2.55 mm, respectively. Therapeutic ultrasound achieved significantly greater pain reduction (*p* = 0.002) and greater improvement in mouth opening (*p* = 0.032). No adverse events were reported.

**Conclusion:**

Both treatments were effective for muscular temporomandibular disorders; however, therapeutic ultrasound was associated with greater short-term improvements in pain intensity and maximum mouth opening than pharmacotherapy under the conditions of this study.

**Clinical Trial Registration:**

https://ctri.nic.in/Clinicaltrials/login.php, identifier CTRI/2024/12/077788.

## 1 Introduction

Temporomandibular disorders (TMDs) comprise a heterogeneous group of musculoskeletal and neuromuscular conditions affecting the temporomandibular joint (TMJ), masticatory muscles, and associated structures. They represent one of the most common causes of chronic non-odontogenic orofacial pain and functional limitation, frequently impairing mastication, speech, and quality of life (1–4).

The global prevalence of TMDs has been estimated to be approximately 31% among adults and 11% among adolescents, with muscular disorders accounting for nearly 45% of all TMDs (5). **Myofascial pain** is the most common type of masticatory muscle disorder which typically presents as a dull aching pain with tender spots on muscles which may even radiate as ear pain and toothache, muscle tightness, and limited mouth opening (6, 7). The Masseter and Temporalis are the most commonly affected muscles (5).

Pain relief, normal jaw function, and maintaining a normal way of life are the primary treatment goals. The individual's needs are considered while deciding a treatment plan. There are multiple treatment modalities, including conservative therapy, physiotherapy, behavioural therapy, occlusal appliances, and pharmacotherapy (7). Pharmacotherapy commonly consists of non-steroidal anti-inflammatory drugs (NSAIDs), analgesics, muscle relaxants, and, in selected cases, antidepressants or anxiolytics (8). Although pharmacotherapy effectively reduces pain, prolonged use may be associated with gastrointestinal, renal, cardiovascular, and central nervous system adverse effects (9). Therapeutic ultrasound, Transcutaneous electrical nerve stimulation, low-level laser application and microwave therapy are electrophysical type of treatment (7). These modalities act by reducing pain by decreasing inflammation, reducing the musculoskeletal load, facilitating return to normal daily activitiesand restoring normal jaw function (4).

Therapeutic ultrasound has emerged as a non-invasive physiotherapeutic modality that uses sound waves at frequencies higher than what the human ear can hear. In clinical settings, it typically operates at frequencies between 1 and 3 MHz, and the intensities used are between 1 and 3 W/cm2 (10) which is capable of producing both thermal and non-thermal effects. Thermal effects increase local tissue temperature, blood flow, collagen extensibility, and muscle relaxation, whereas non-thermal mechanisms, including stable cavitation and acoustic microstreaming, enhance cellular permeability, tissue repair, angiogenesis, and collagen synthesis (11, 12). These mechanisms collectively contribute to pain reduction, improved tissue healing, and restoration of mandibular function. Although several randomized clinical trials have demonstrated beneficial effects of therapeutic ultrasound in reducing pain and improving mouth opening among patients with muscular TMDs, comparative evidence directly evaluating therapeutic ultrasound against conventional pharmacotherapy remains limited (13–21). Therefore, the present randomized clinical trial aimed to compare the effectiveness of therapeutic ultrasound and pharmacotherapy in reducing pain intensity and improving maximum mouth opening in patients with muscular temporomandibular disorders.

## 2 Materials and methods

### 2.1 Study design

This prospective, parallel-group randomized clinical trial with a 1:1 allocation ratio was conducted to compare the effectiveness of therapeutic ultrasound and pharmacotherapy in the management of muscular temporomandibular disorders (TMDs). The study was designed and reported in accordance with the Consolidated Standards of Reporting Trials (CONSORT) 2010 Statement. The study protocol was approved by the Institutional Scientific Review Board prior to commencement and was prospectively registered with the Clinical Trials Registry–India (CTRI).

### 2.2 Ethical approval and trial registration

The study was conducted in accordance with the ethical principles of the Declaration of Helsinki. Ethical approval was obtained from the Institutional Ethics Committee of Yenepoya (Deemed to be University), Mangalore, India (Approval No. **YEC-1/2024/038**). The trial was prospectively registered with the Clinical Trials Registry–India (CTRI; Registration No. **CTRI/2024/12/077788**; registered on **06 December 2024**). Written informed consent was obtained from all participants before enrolment into the study.

### 2.3 Study setting

The study was conducted in the Department of Oral Medicine and Radiology, Yenepoya Dental College, Yenepoya (Deemed to be University), Mangalore, Karnataka, India, between December 2024 and January 2026**.** Eligible participants attending the outpatient department with symptoms suggestive of muscular temporomandibular disorders were screened for recruitment.

### 2.4 Participants

**Figure 1:**
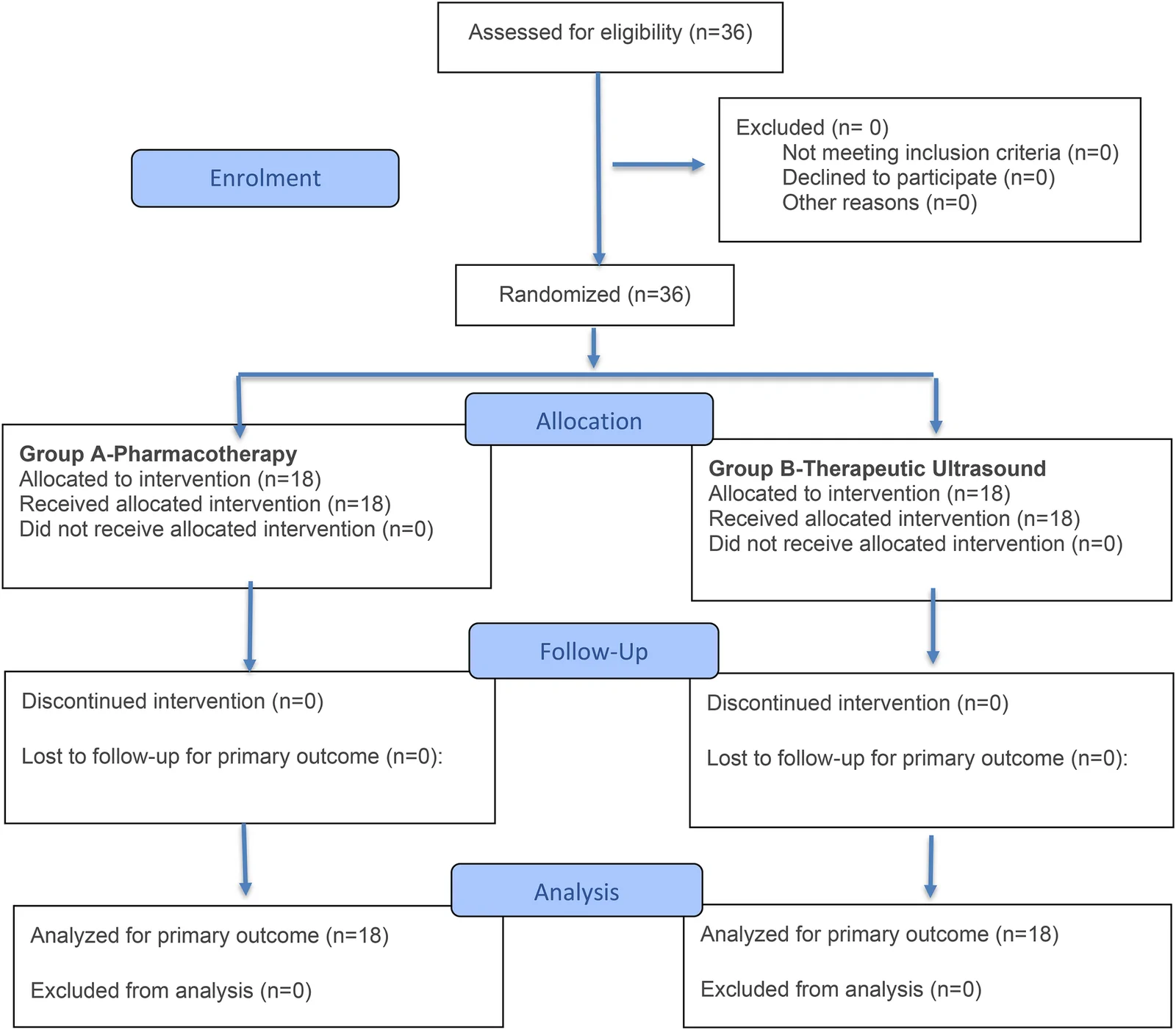
CONSORT flow diagram.

Patients diagnosed with muscular temporomandibular disorders according to **Group I of the Research Diagnostic Criteria for Temporomandibular Disorders (RDC/TMD)** were assessed for eligibility. RDC/TMD was selected because it provides standardized and validated diagnostic criteria for Group I (muscular) TMDs and has been widely used in clinical trials evaluating conservative treatment modalities. The diagnostic criteria were predefined in the study protocol before participant recruitment and were applied consistently throughout the study. A total of **36 participants** fulfilling the eligibility criteria were recruited and randomly allocated into two equal groups (*n* = 18 each). Participant recruitment, allocation, follow-up, and analysis are presented in the CONSORT flow diagram (Figure 1).

### 2.5 Eligibility criteria

#### 2.5.1 Inclusion criteria

Included patients aged 16–60 years diagnosed with muscular TMDs based on Group I of the Research Diagnostic Criteria for TMD, including myofascial pain, and myofascial pain with limited opening.

- **Myofascial pain:**
  1. Reported pain in masticatory muscles
  2. Pain on palpation in at least 3 sites, one of them at least in the same side of the reported pain
- **Myofascial pain with limited mouth opening:**
  1. Myofascial pain
  2. Pain-free unassisted opening < 40 mm and Passive stretch ≥ 5 mm

#### 2.5.2 Exclusion criteria

Included patients with history of trauma/fracture, Orofacial infection, Skin lesion, Pregnant women, Benign and malignant lesions of TMJ, Cardiac pacemakers, arrhythmias, kidney diseases, and allergy to acoustic gel.

### 2.6 Sample size calculation

The sample size was calculated using G*Power software version 3.1.9.4 based on the findings of **Shalu Rai et al.** An effect size of **1.126**, a power of **90%**, and a significance level (α) of **0.05** were considered. The minimum required sample size was calculated as **36 participants**, with **18 participants allocated to each treatment group**.

### 2.7 Randomization and allocation concealment

Participants were randomly assigned to either the pharmacotherapy group (Group A) or the therapeutic ultrasound group (Group B) using a computer-generated random number sequence with a 1:1 allocation ratio. The principal investigator generated the randomization sequence, enrolled eligible participants, and assigned them to the respective treatment groups according to the computer-generated allocation list. Allocation concealment was not implemented. Owing to the nature of the interventions, blinding of participants and the treating investigator was not feasible.

#### 2.7.1 Intervention

Participants underwent a comprehensive examination of the temporomandibular joint, muscles of mastication, and occlusion, following which patients having pain associated with TMDs of muscular origin were randomly allocated into either of the two groups i.e., GROUP A and GROUP B.

#### 2.7.2 Group A: pharmacotherapy

Participants allocated to Group A received pharmacotherapy consisting of **Ibuprofen (400 mg), Paracetamol (325 mg), and Chlorzoxazone (250 mg)** administered orally **twice daily for seven consecutive days**. This combination was selected because it combines anti-inflammatory, analgesic, and muscle-relaxant actions, thereby addressing pain, inflammation, and muscle spasm associated with muscular TMD. This combination represents a commonly used conservative treatment approach for myogenous temporomandibular disorders. Participants were advised regarding drug compliance and were instructed to report any adverse effects during the study period.

#### 2.7.3 Group B: therapeutic ultrasound

**Figure 2:**
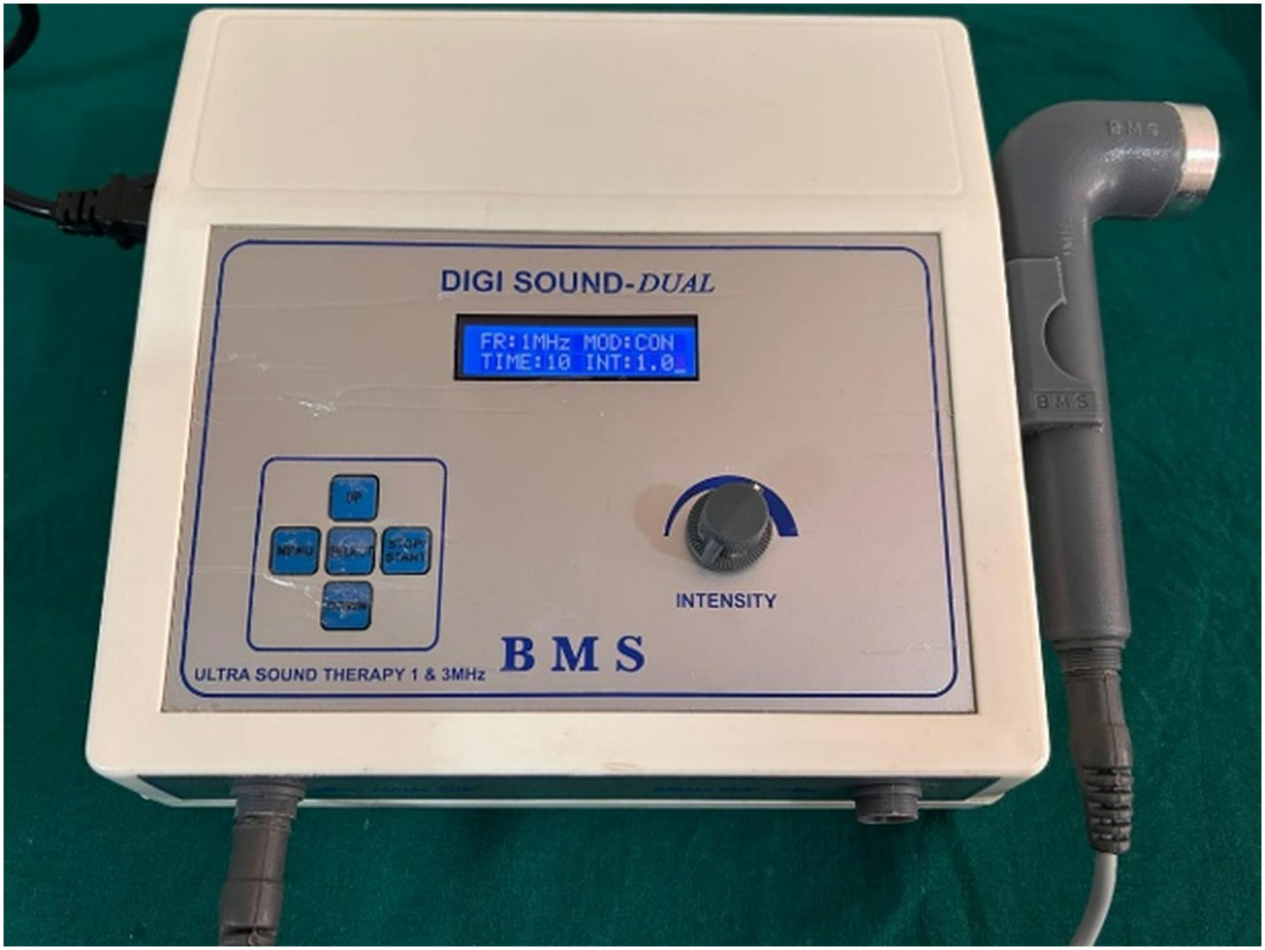
Therapeutic ultrasound with the parameters.

**Figure 3:**
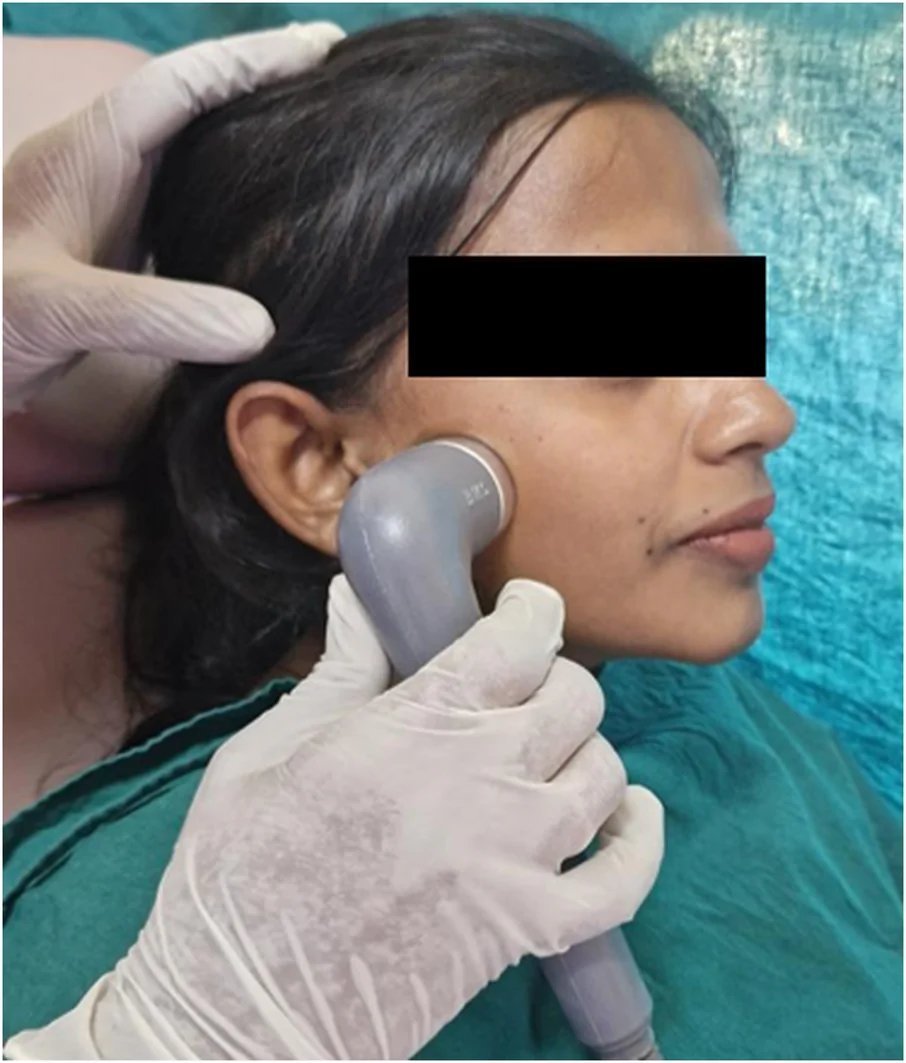
Participant during ultrasound therapy.

Participants allocated to Group B received therapeutic ultrasound using portable therapeutic ultrasound [DIGI SOUND- DUAL] at a frequency of **1 MHz**, an intensity of **1.0 W/cm²**, in **continuous mode**, for **10 min per session**, once daily for **seven consecutive working days**. The parameters were selected based on previously published clinical studies demonstrating its effectiveness in reducing pain and improving mandibular function in patients with muscular TMD while maintaining a practical treatment duration for routine clinical practice. The procedure involved application of acoustic gel over the affected muscles, after which the ultrasound transducer was moved continuously in slow circular motions with gentle pressure to ensure uniform energy distribution and avoid periosteal overheating. All treatment sessions were administered by the same investigator using a standardized treatment protocol (Figures 2, 3).

### 2.8 Outcome measures

#### 2.8.1 Primary outcome

The primary outcome was **pain intensity**, assessed using the **Numerical Rating Scale (NRS)**, where 0 represented “no pain” and 10 represented “the worst imaginable pain.”

#### 2.8.2 Secondary outcome

**Figure 4:**
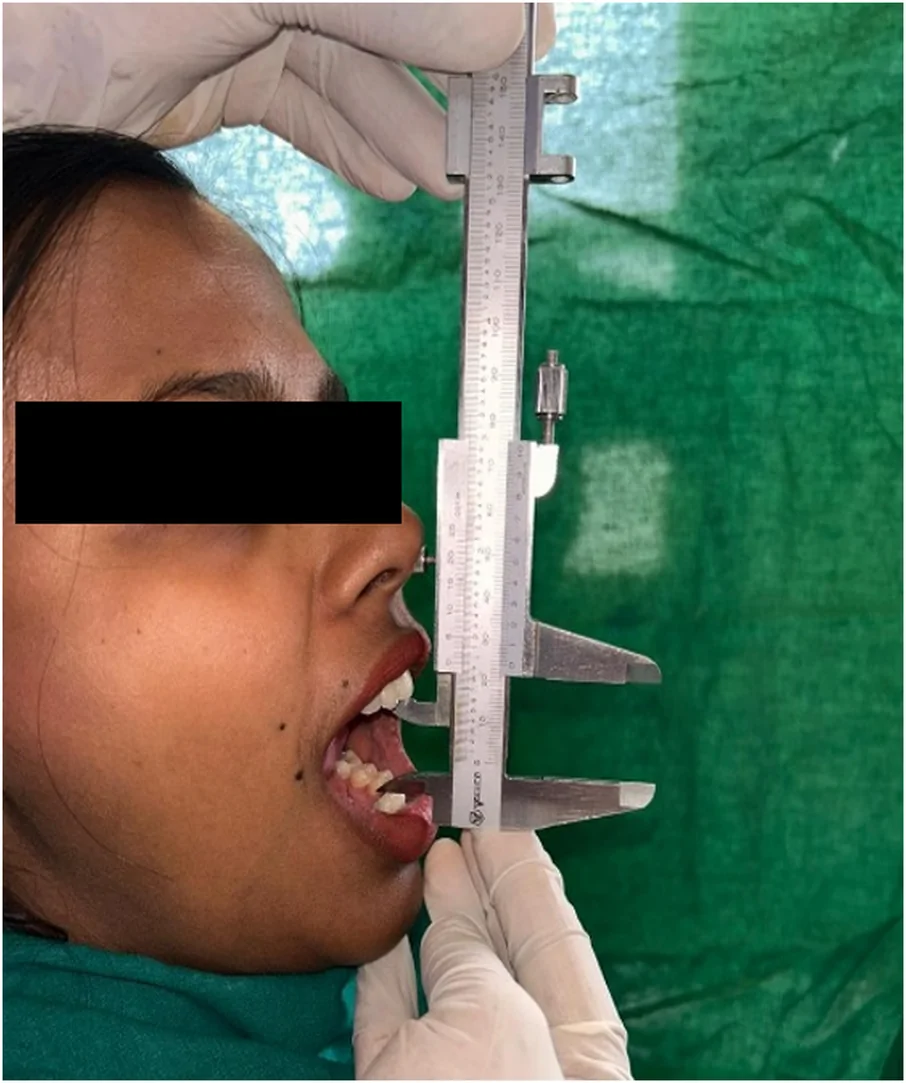
Measuring maximum mouth opening.

The secondary outcome was **maximum mouth opening (MMO)**, measured in millimetres using a calibrated Vernier calliper as the distance between the incisal edges of the maxillary and mandibular central incisors during maximum unassisted mouth opening (Figure 4).

Clinical assessments were performed at **baseline (Day 0)** and **post-treatment (Day 7)** by the same examiner using standardized procedures. Telephonic follow-up was conducted one month after completion of treatment.

### 2.9 Statistical analysis

Data were analysed using **Jamovi software version 2.6.44**. Continuous variables were presented as **mean ± standard deviation (SD)**. Normality of data distribution was assessed using the **Shapiro–Wilk test**. Intra-group comparisons were performed using the **paired Student’s t-test**, whereas inter-group comparisons were analysed using the **independent Student's t-test**. A two-tailed *p*-value of **<0.05** was considered statistically significant.

## 3 Results

### 3.1 Participant flow

A total of **36 participants** who fulfilled the eligibility criteria were enrolled in the study and randomly allocated to either the pharmacotherapy group (Group A, *n* = 18) or the therapeutic ultrasound group (Group B, *n* = 18). All participants completed the allocated intervention and post-treatment evaluation. No participants were lost to follow-up or excluded from the final analysis. The participant recruitment, allocation, follow-up, and analysis are illustrated in the CONSORT flow diagram (Figure 1).

### 3.2 Baseline demographic and clinical characteristics

The study included participants aged **16 to 55 years**. The mean age of participants in Group A was **32.72** **±** **12.40 years**, while that of Group B was **35.11** **±** **13.14 years**, with no statistically significant difference between the groups (*p* = 0.54). Group A consisted of **8 males and 10 females**, whereas Group B comprised **9 males and 9 females**. The baseline maximum mouth opening was **40.31** **±** **3.63 mm** in Group A and **40.75** **±** **3.55 mm** in Group B, with no statistically significant difference between the groups (*p* = 0.733). These findings indicate that both groups were comparable at baseline (Table 1).

**Table 1:** Baseline background of the study participants.

Variables	Group A (*n* = 18)	Group B (*n* = 18)
Age distribution (years)		
Minimum	18	16
Maximum	54	55
Mean ± SD	32.7 ± 12.4	35.1 ± 13.1
Gender distribution, *n* (%)		
Male	8 (44.4)	9 (50)
Female	10 (55.6)	9 (50)

### 3.3 Pain intensity

Both treatment groups demonstrated a statistically significant reduction in pain intensity following the intervention.

In Group A (pharmacotherapy), the mean Numerical Rating Scale (NRS) score decreased from **6.31** **±** **1.35** at baseline to **2.94** **±** **1.06** after treatment, corresponding to a mean reduction of **3.37 points** (*p* < 0.001).

In Group B (therapeutic ultrasound), the mean NRS score decreased from **5.56** **±** **1.97** at baseline to **1.69** **±** **1.08** after treatment, representing a mean reduction of **3.87 points** (*p* < 0.001).

Inter-group comparison of post-treatment pain scores demonstrated significantly lower pain intensity in the therapeutic ultrasound group compared with the pharmacotherapy group (**1.69** **±** **1.08 vs. 2.94** **±** **1.06**, *p* = 0.002), indicating superior pain reduction following therapeutic ultrasound (Table 2).

**Table 2:** Numerical rating scale (NRS) pain scores at baseline and post-intervention.

Time point	Group A Mean ± SD	Group B Mean ± SD	*p*-value
Baseline	6.31 ± 1.35	5.56 ± 1.97	0.218
Post-treatment	2.94 ± 1.06	1.69 ± 1.08	**0.002** ^a^

Bold value indicates a highly statistically significant difference (*p* < 0.01).

a highly statistically significant.

### 3.4 Maximum mouth opening

Both treatment groups demonstrated significant improvement in maximum mouth opening following treatment.

In Group A, the mean maximum mouth opening increased from **40.31** **±** **3.63 mm** at baseline to **41.44** **±** **2.94 mm** after treatment, representing a mean increase of **1.13 mm** (*p* = 0.006).

In Group B, the mean maximum mouth opening increased from **40.75** **±** **3.55 mm** at baseline to **43.63** **±** **2.55 mm** after treatment, representing a mean increase of **2.88 mm** (*p* < 0.001).

Comparison of post-treatment values demonstrated significantly greater improvement in maximum mouth opening in the therapeutic ultrasound group than in the pharmacotherapy group (**43.63** **±** **2.55 mm vs. 41.44** **±** **2.94 mm**, *p* = 0.032) (Table 3).

**Table 3:** Mouth opening measurements (mm) at baseline and post-intervention.

Time point	Group A Mean ± SD	Group B Mean ± SD	*p*-value
Baseline	40.31 ± 3.63	40.75 ± 3.55	0.733
Post-treatment	41.44 ± 2.94	43.63 ± 2.55	**0.032** ^a^

Bold value indicates a statistically significant difference (*p* < 0.05).

a statistically significant.

### 3.5 Harms

No harms or adverse events were observed in either group during the study period.

## 4 Discussion

Temporomandibular disorders (TMDs) of muscular origin are a common cause of orofacial pain and functional limitation (1). Pain relief and restoration of normal jaw function remain the primary goals of management (7). In the present study, the majority of patients belonged to the third and fourth decades of life. This finding is consistent with the observations of Ouanounou et al. (2017) (22) and Kalladka et al. (2021) (23), who reported that muscular TMDs are more prevalent among individuals aged 20–40 years, possibly due to increased psychological stress, parafunctional habits, and greater functional demands on the masticatory system (22, 23).

With regard to gender distribution, a slight female predominance was observed in the present study. Similar findings have been reported by Minervini et al. (2024) (24) and Al-Moraissi et al. (2022) (25), who suggested that this predominance may be attributed to psychosocial factors, hormonal influences, and increased pain sensitivity among females (24, 25).

In Group A, pharmacotherapy resulted in a 50% reduction in pain intensity, which was highly statistically significant (*p* < 0.001). These findings are in agreement with those reported by Herman et al. (2002) (26), who demonstrated significant pain reduction following the use of muscle relaxants in patients with muscular TMD (26).

Similarly, maximum mouth opening showed a mean increase of 1.13 mm. Although this improvement was statistically significant (*p* = 0.006), the magnitude of functional improvement was relatively modest. Comparable observations were reported by Kulkarni et al. (2020) (27) and Christidis et al. (2023), who concluded that non-steroidal anti-inflammatory drugs (NSAIDs) provide effective symptomatic pain relief in patients with TMDs but may not consistently produce substantial improvements in jaw function. They also emphasized that the available long-term evidence supporting sustained functional benefits remains limited. Pharmacotherapy primarily acts by reducing inflammatory mediators and relieving muscle spasm, thereby decreasing pain; however, it has limited direct effects on muscle flexibility and tissue remodeling (9, 27).

In Group B, therapeutic ultrasound produced a 69.6% reduction in pain intensity, demonstrating its effectiveness in significantly alleviating pain and interrupting the pain-spasm cycle. This reduction was highly statistically significant (*p* < 0.001). Regarding functional improvement, maximum mouth opening increased by a mean of 2.88 mm, which was also highly statistically significant (*p* < 0.001).

These findings are consistent with those reported by Shuang Ba et al. (2021), who demonstrated significant improvements in pain intensity and mandibular movements following therapeutic ultrasound. Similarly, Mishra et al. (2022) (7) reported statistically significant reductions in pain along with increased mouth opening, while Rai et al. (2016) (14) observed greater subjective and sonographic improvement in patients treated with therapeutic ultrasound compared with transcutaneous electrical nerve stimulation (TENS) (1, 7, 14).

The post-treatment intergroup comparison demonstrated a statistically significant improvement in mouth opening in the therapeutic ultrasound group compared with the pharmacotherapy group. Likewise, pain intensity was significantly lower in the ultrasound group, indicating greater analgesic efficacy than pharmacotherapy.

Within the limitations of the present short-term randomized clinical trial, therapeutic ultrasound demonstrated greater improvements in pain intensity and maximum mouth opening than pharmacotherapy. While pharmacotherapy primarily provides symptomatic relief by reducing inflammation and muscle spasm, it does not directly improve tissue extensibility or local muscle biomechanics, resulting in comparatively limited improvement in mouth opening. In contrast, therapeutic ultrasound acts directly on the affected muscle tissues by enhancing local microcirculation, reducing muscle stiffness, increasing collagen extensibility, promoting tissue healing and cellular repair, and reducing inflammation without the systemic adverse effects associated with medications. These mechanisms may explain the superior pain relief and functional outcomes observed in Group B (11, 12).

The findings of the present study are also consistent with recent systematic reviews and network meta-analyses evaluating conservative management of myogenous temporomandibular disorders. Al-Moraissi et al. reported that conservative treatment modalities, including physiotherapeutic interventions, are effective in reducing pain and improving mandibular function, although no single modality demonstrated consistent superiority across all clinical outcomes (25). Similarly, Christidis et al. concluded that pharmacological therapies provide short-term symptomatic relief, while emphasizing the limited quality of evidence supporting sustained long-term benefits (9). Furthermore, Minervini et al. emphasized that conservative management of myogenous TMD is most effective when individualized and delivered as part of a multimodal treatment approach rather than relying on a single intervention (24). In agreement with these findings, both interventions evaluated in the present study resulted in significant clinical improvement; however, therapeutic ultrasound demonstrated greater short-term reductions in pain intensity and improvements in maximum mouth opening than pharmacotherapy. These findings support the potential role of therapeutic ultrasound as a promising conservative treatment modality while highlighting the need for larger, high-quality randomized controlled trials with longer follow-up periods to confirm its long-term clinical effectiveness.

Although telephonic follow-up was conducted one month after completion of treatment to assess the patient's clinical status which showed the subjective assessment of efficacy of the modalities remained the same, however objective assessment could not be performed. Therefore, the long-term effectiveness and sustainability of the treatment outcomes could not be objectively evaluated.

The strengths of the present study include its randomized controlled design, the use of standardized participant selection using RDC/TMD diagnostic criteria, validated subjective [Numerical Rating Scale (NRS)] and objective (maximum mouth opening measured using a Vernier calliper) outcome measures, standardized treatment protocols, and comparable baseline characteristics between the study groups, thereby enhancing the internal validity of the findings. Although DC/TMD is currently the recommended international diagnostic standard, RDC/TMD remains a validated and widely accepted diagnostic system for research involving myogenous TMDs. Its use in the present study ensured standardized participant selection and facilitated comparison with previously published randomized clinical trials evaluating conservative therapies (3). Furthermore, the study evaluated therapeutic ultrasound as a non-invasive, safe, and clinically relevant treatment modality for the management of muscular TMD.

The limitations of the present study include a relatively small sample size, a short follow-up period restricted to immediate post-treatment evaluation, and the inability to objectively assess long-term recurrence or sustained treatment outcomes, the absence of allocation concealment may have introduced selection bias, while the absence of blinded outcome assessment may have resulted in performance and assessment bias. Furthermore, the lack of a sham ultrasound control group and the difference in treatment delivery between the study groups may have influenced the observed outcomes.

Further multicentric randomized controlled trials with larger sample sizes and extended follow-up periods are recommended to validate these findings and evaluate the long-term effectiveness, durability of treatment outcomes, and recurrence rates associated with therapeutic ultrasound in the management of muscular temporomandibular disorders. Future studies should incorporate equivalent clinician contact or supervised interventions in both treatment groups, implement allocation concealment and blinded outcome assessment wherever feasible, and include a sham ultrasound control group to minimize bias and better differentiate the specific therapeutic effects of ultrasound from placebo responses.

## 5 Conclusion

Therapeutic ultrasound was associated with greater short-term improvements in pain intensity and maximum mouth opening than pharmacotherapy in patients with muscular temporomandibular disorders under the conditions of this study. After seven days of treatment, therapeutic ultrasound produced significantly greater improvements in pain reduction and functional outcomes compared with pharmacotherapy. These findings suggest that therapeutic ultrasound may be a safe, non-invasive conservative treatment option and a potential alternative or adjunct to pharmacotherapy, particularly for patients in whom prolonged medication use may be undesirable.

## Data Availability

The raw data supporting the conclusions of this article will be made available by the authors, without undue reservation.
